# A Randomized Trial to Compare Ultrasound-Guided Dorsalis Pedis Versus Posterior Tibial Artery Cannulation in Neurosurgical Patients

**DOI:** 10.7759/cureus.33514

**Published:** 2023-01-08

**Authors:** Ashutosh Kaushal, Nirupa Ramakumar, Praveen Talawar, Priyanka Gupta, Vaishali Waindeskar, Anuj Jain, Sunaina T Karna, Sweta Kumari

**Affiliations:** 1 Anesthesiology, All India Institute of Medical Sciences, Bhopal, Bhopal, IND; 2 Anesthesiology, All India Institute of Medical Sciences, Rishikesh, Rishikesh, IND; 3 Microbiology, All India Institute of Medical Sciences, Bhopal, Bhopal, IND

**Keywords:** neurosurgery, arterial cannulation, posterior tibial artery, dorsalis pedis artery, ultrasound

## Abstract

Introduction

Dorsalis pedis or posterior tibial artery is selected as an alternative to radial artery cannulation when there is no access or unsuccessful cannulation of a radial artery. This study aimed to compare the two major arteries of the foot (dorsalis pedis and posterior tibial) in terms of their ultrasound (USG)-guided cannulation characteristics in patients posted for elective neurosurgical procedures.

Methods

All consenting patients, 18-65 years of age, scheduled for elective neurosurgical procedures under general anesthesia requiring arterial cannulations were enrolled. The first-pass success rate, assessment time, cannulation time, total procedural time, and the number of cannulation attempts for both procedures were estimated.

Results

A total of 90 patients were included in the study. The assessment time, cannulation time, and total time for arterial cannulation were significantly greater in the dorsalis pedis artery group than in the posterior tibial artery group (p < 0.001). Successful arterial cannulation in the first attempt was 73.3% in the dorsalis pedis, whereas it was 80% in the posterior tibial group but comparable (p = 0.455). The successful cannulation outcome was slightly more in the posterior tibial artery group but comparable (p = 1.00).

Conclusion

First-pass successful cannulation rates in the posterior tibial and the dorsalis pedis artery are comparable. However, the assessment time, cannulation time, and total procedural time are higher and statistically significant for dorsalis pedis artery cannulation compared to the posterior tibial artery group.

## Introduction

Intraarterial cannulation is commonly instituted to monitor continuous invasive blood pressure and cardiac output, analyze frequent arterial blood gas, assess fluid responsiveness, and guide fluid therapy intraoperatively and in the intensive care unit [1,2].

The common sites for arterial placement are the radial, ulnar, brachial, axillary, posterior tibial, dorsalis pedis, and femoral arteries [3,4]. Because of its proximity to the skin surface, collateral circulation with the ulnar artery, and low complication rate, the radial artery is most preferred for arterial cannulation among these potential sites [5].

Dorsalis pedis artery or posterior tibial artery is selected as an alternative to the radial artery cannulation in cases of inaccessibility or unsuccessful cannulation of a radial artery due to anatomical abnormalities/tortuosity and low pulse volume, in head and neck surgeries if the upper limb is burnt or injured or in surgical procedures involving arms [6,7].

The ultrasound (USG) technique has become a standard of care due to the real-time visualization of landmarks, reduced complications, less time spent at the bedside, and improved success rates [8,9]. In small children, the posterior tibial artery is a reasonable alternative to the radial artery for USG-guided arterial cannulation [10].

There is a literature deficit regarding the comparison of dorsalis pedis artery and posterior tibial artery cannulation using USG guidance. Therefore, this study aimed to find the preferred foot artery (dorsalis pedis or posterior tibial) while attempting USG-guided arterial cannulation.

The primary objective of this study was to compare the USG-guided first-pass success rate of posterior tibial versus dorsal pedis artery cannulation in adult patients undergoing neurosurgery under general anesthesia. The secondary objectives were to compare assessment time, cannulation time, total procedural time, and the number of cannulation attempts for the posterior tibial and dorsalis pedis arteries.

## Materials and methods

This prospective randomized control study was conducted at a tertiary care research medical institute from October 2020 to March 2021 after obtaining approval from the Institutional Ethics Committee (AIIMS/IEC/20/626, dated 12/09/2020). The trial was registered under the clinical trial registry in India (CTRI/2020/10/028248). Written informed consent was taken from the patient after a thorough explanation of the purpose and procedure of the study.

All consenting patients of either gender, between the ages of 18 and 65, who met the American Society of Anesthesiologists Physical Status I & II scheduled for elective neurosurgeries under general anesthesia requiring arterial cannulation were enrolled. Patients with no posterior tibial or dorsalis pedis arterial pulsation and skin erosions near the insertion site, patients undergoing surgery on lower limbs, patients with peripheral vascular disease and coagulopathies, and patients with body mass index > 30 kg/m^2^ were excluded.

A study by Kim et al. reported that children's first-pass success rate for posterior tibial artery and dorsalis pedis artery cannulation was 75% and 45%, respectively [10]. Assuming 80% power and a 95% confidence interval, the sample size required was 42 patients per arm. Accepting 5% dropout in each group, 45 patients in each arm were recruited.

The enrolled patients were randomized to either group based on a sequence of computer-generated random numbers. The serially-numbered sealed opaque envelope method was used for allocation concealment, and the envelope was opened just before the procedure to reveal allocation. The anesthesiologist assigned to the intervention was aware of the group allocation during the arterial cannulation. An unblinded anesthesiologist who was not part of the study collected the primary and secondary data. The data analyst and the outcome accessor were unaware of the identity of the study groups.

Once the patient was shifted to the operation room, after induction of general anesthesia and tracheal intubation with standard protocol, arterial cannulation was planned. The arterial cannulation was performed just after intubation in both groups.

All the cannulations were performed by a single investigator who had performed more than 100 USG-guided cannulations of the dorsalis pedis and posterior tibial arteries to minimize inter-individual variability. After taking all aseptic precautions, the ankle was dorsiflexed up to 90 degrees, and eversion of the foot was done in case of the posterior tibial artery and neutral position for dorsalis pedis artery cannulation.

The USG (SonoSite M- Turbo, Fujifilm Corporation, Tokyo, Japan) vascular linear probe footprint (6-13 MHz) was placed in the groove between the medial malleolus and Achilles tendon in the case of posterior tibial artery cannulation. In the case of dorsalis pedis artery cannulation, the USG probe was kept at the dorsum of the foot. In either group, after viewing the artery in cross-section, the transducer was rotated 90°, keeping the image in the center of the USG screen to identify the artery in its long axis. A 20G arterial cannula (Becton, Dickinson and Company, New Jersey, USA) was inserted under the center of the transducer by an in-plane approach. Mean arterial pressure and heart rate were recorded just before arterial cannulation in both groups.

The time from the beginning of screening the artery to skin penetration by the needle was defined as assessment time. The cannulation time was defined as the time from the initial skin puncture to the successful placement of the cannula in the artery. Successful arterial cannulation was defined as confirmation of the continuation of a gush of arterial blood and arterial waveform after cannulation. Total time was defined as the summation of assessment time and cannulation time. It started with placing a USG probe over the area of interest and ended with successful cannulation of the artery (confirmation of arterial waveform after cannulation). The total number of attempts for successful arterial cannulation was documented. Unsuccessful arterial cannulation after three attempts was defined as a failure, whereas the success rate was determined as successful arterial cannulation in a maximum of three attempts [7]. After three attempts, an alternative site was chosen for arterial cannulation. Adverse effects, if any, related to arterial cannulation, like thrombosis, hematoma, local swelling, tenderness, ischemia distal to the insertion site, or distal discoloration, were monitored until the day following decannulation.

Statistical analysis was performed using Statistical Package for Social Sciences (SPSS Inc., IBM Corp., Armonk, NY) version 22.0. Continuous variables were expressed as mean and standard deviation. Categorical variables were expressed in number and percentage. The variables such as age, body mass index, total time for cannulation, mean arterial pressure, and heart rate were normally distributed; thus, parametric tests (t-tests) were used to make group comparisons. The variables such as assessment time (sec) and cannulation time (sec) were not normally distributed; thus, non-parametric tests (Wilcoxon-Mann-Whitney U test) were used to make group comparisons. Chi-squared test was used to explore the association between the group with first-pass success. Fisher's exact test was used to examine the association between the group with the number of attempts, cannulation outcome, and total time as more than 20% of the total number of cells had an expected count of less than 5.

## Results

**Figure 1:**
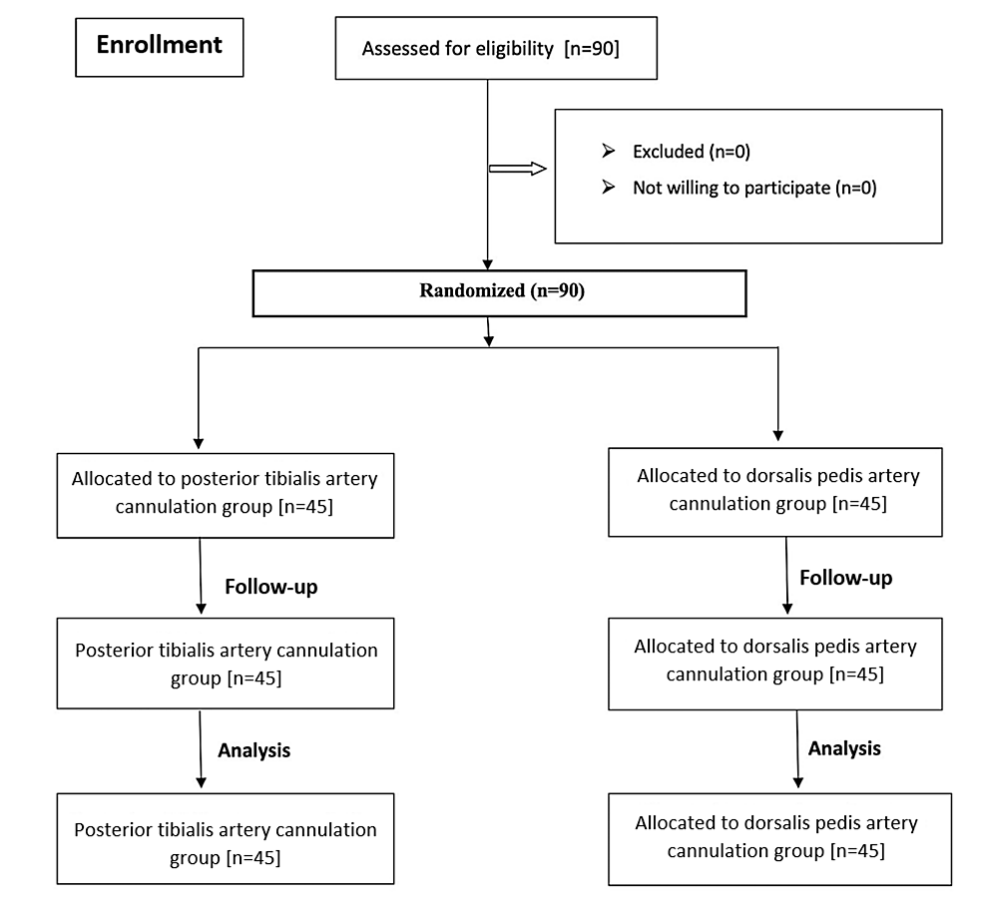
Consolidated Standards of Reporting Trials (CONSORT) flowchart

A total of 90 patients were included in the study. The Consolidated Standards of Reporting Trials (CONSORT) flowchart is shown in Figure 1.

The distribution of baseline demographic parameters, mean arterial pressure, and heart rate at the time of arterial cannulation was comparable between the groups and is depicted in Table 1.

**Table 1:** Comparison of baseline demographic parameters of the patients ^1^t-test; ^2^Chi-squared test. n: Number; SD: Standard deviation.

Parameters		Group		p-value
		Dorsalis pedis artery (n = 45)	Posterior tibial artery (n = 45)	
Age (Years), Mean ± SD		45.73 ± 16.91	42.24 ± 15.36	0.308^1^
Gender, n (%)	Male	30 (66.7%)	26 (57.8%)	0.384^2^
	Female	15 (33.3%)	19 (42.2%)	
Body mass index (kg/m²), Mean ± SD		23.01 ± 1.97	22.64 ± 2.38	0.423^1^
Mean arterial pressure (mm/Hg), Mean ± SD		89.11 ± 6.59	89.36 ± 8.07	0.875^1^
Heart rate (beat per minute), Mean ± SD		75.84 ± 10.96	76.24 ± 12.98	0.875^1^

The first pass success, assessment time, cannulation time, total time, number of attempts, and cannulation outcome are depicted in Table 2.

**Table 2:** Comparison of cannulation characteristics in two groups ^ 1^t-test; ^2^Chi-squared test; ^3^Wilcoxon-Mann-Whitney U test;^ 4^Fisher's exact test. n: Number; SD: Standard deviation.

Parameters		Group		p-value
		Dorsalis pedis artery (n = 45)	Posterior tibial artery (n = 45)	
Assessment time (seconds), Mean ± SD		27.36 ± 4.28	17.56 ± 4.31	<0.001^3^
Cannulation time (seconds), Mean ± SD		19.84 ± 1.58	13.58 ± 1.60	<0.001^3^
Total time (seconds), Mean ± SD		47.20 ± 4.41	31.18 ± 4.80	<0.001^1^
Number of attempts, n (%)	1	33 (73.3%)	36 (80.0%)	0.850^4^
	2	6 (13.3%)	5 (11.1%)	
	3	3 (6.7%)	2 (4.4%)	
	>3	3 (6.7%)	2 (4.4%)	
First-pass success (Yes), n (%)		33 (73.3%)	36 (80.0%)	0.455^2^
Cannulation outcome	Success	42 (93.3%)	43 (95.6%)	1.000^4^

The assessment time, cannulation time, and total time for arterial cannulation were significantly greater in the dorsalis pedis artery group than in the posterior tibial artery group (p < 0.001). Successful arterial cannulation in the first attempt was more in the posterior tibial artery group than the dorsalis pedis artery group and was comparable (p = 0.455). Successful arterial cannulation in the second, third, and more than the third attempts was more in the dorsalis pedis artery group than the posterior tibial artery group and was comparable (p = 0.850). The successful cannulation outcome was slightly more in the posterior tibial artery group but was comparable (p = 1.00). In both groups, patients do not develop any adverse effects related to arterial cannulation.

## Discussion

The posterior tibial and dorsalis pedis are the principal arteries of the foot. The posterior tibial artery originates at the level of the lower border of the popliteus muscle as one of the terminal divisions of the popliteal artery. It passes between the medial malleolus and the Achilles tendon [11].

The dorsalis pedis artery originates as a continuation of the anterior tibial artery in front of the ankle joint. It passes midway between the medial and lateral malleoli till the proximal end of the first dorsal intermetatarsal space. Then it goes inferiorly as the deep plantar artery to complete the plantar arch in the sole with the lateral plantar branch of the posterior tibial artery [12].

The posterior tibial and dorsalis pedis arteries are often considered alternatives to radial arteries for arterial cannulation by the palpatory method. However, a systematic review and meta-analysis suggested using ultrasound as an adjunct to arterial cannulation [13].

In the present study, though the results were not clinically significant, there was a 95.6% success rate for the posterior tibial artery cannulation compared to the 93.3% success rate for the dorsalis pedis artery cannulation. The assessment time, cannulation time, and total procedural time were significantly greater for the USG-guided dorsalis pedis than for the posterior tibial artery cannulation.

Kim et al. compared the initial success rate of USG-guided arterial cannulation among three arteries in small children and found that the first-attempt success rate of the posterior tibial artery (75%) was significantly higher than that of the dorsalis pedis artery (45%) [10].

They found that the median cannulation time of the posterior tibial artery was shorter than that of the dorsalis pedis artery. In contrast, the cross-sectional area and depth from the skin surface of the posterior tibial artery were significantly greater than the dorsalis pedis artery. However, no study compared the USG-guided posterior tibial artery with the dorsalis pedis artery in adults.

The large diameter, deep location from the skin surface, and ease of USG probe placement in the groove between the medial malleolus and the Achilles tendon could be the probable favorable factors for cannulation of the posterior tibial artery when compared to the dorsalis pedis arteries.

As the dorsalis pedis artery is more superficial, there is the possibility of arterial compression due to the weight of the USG probe. In contrast, the deeper location of the posterior tibial artery makes the artery noncompressible when the USG probe is manipulated [10].

In previous studies, the diameter of the posterior tibial artery was found to be greater than the dorsalis pedis artery in adults [14,15]. Also, the anatomical position makes it challenging to bring the dorsalis pedis artery to a horizontal frame during USG-guided assessment. Hence, assessment time might be greater for the dorsalis pedis artery than for the posterior tibial artery. The larger diameter of the posterior tibial artery might have several advantages, including an increased cannulation success rate and fewer complications. However, patients do not develop any adverse effects related to arterial cannulation in both groups.

One of the limitations of the present study is that our results cannot be generalized to the children in whom any advantage of USG for arterial cannulation might be dependent on age [16,17]. The second limitation is that, before arterial cannulation, we did not measure the diameter, flow velocities of the dorsalis pedis artery and posterior tibial artery, and the depth of both arteries from the skin. Although we tried to minimize the ascertainment bias by keeping the data analyst and the outcome accessor unaware of the study groups' identity, the interventionist's awareness of the group allocation during the arterial cannulation is another limitation of this study.

## Conclusions

This study aimed to find the preferred foot artery for cannulation by comparing the USG-guided cannulation characteristics of posterior tibial and dorsalis pedis arteries in adult neurosurgical patients. First-pass successful cannulation in the posterior tibial and the dorsalis pedis arteries is comparable. The assessment time, cannulation time, and total procedural time are higher and statistically significant for dorsalis pedis artery cannulation compared to the posterior tibial artery group. The present study demonstrates that out of the posterior tibial and dorsalis pedis arteries, any artery may be chosen for USG-guided cannulation when first-pass success is considered. However, considering total procedural time, the posterior tibial artery can be selected over the dorsalis pedis artery for USG-guided cannulation.
